# Identification of Multiple Bacteriocins in *Enterococcus* spp. Using an *Enterococcus*-Specific Bacteriocin PCR Array

**DOI:** 10.3390/microorganisms3010001

**Published:** 2015-02-04

**Authors:** Chris Henning, Dhiraj Gautam, Peter Muriana

**Affiliations:** 1 Department of Animal Science, Oklahoma State University, Monroe Street, Stillwater, OK 74078, USA; E-Mail: cdhenni@okstate.edu; 2 Robert M. Kerr Food & Agricultural Products Centre, Oklahoma State University, 109 FAPC Building, Monroe Street, Stillwater, OK 74078-6055, USA; E-Mail: dhiraj.gautam@okstate.edu

**Keywords:** bacteriocin, genes, *Enterococcus*, *Listeria* monocytogenes, food preservation

## Abstract

Twenty-two bacteriocin-producing *Enterococcus* isolates obtained from food and animal sources, and demonstrating activity against *Listeria monocytogenes*, were screened for bacteriocin-related genes using a bacteriocin PCR array based on known enterococcal bacteriocin gene sequences in the NCBI GenBank database. The 22 bacteriocin-positive (Bac+) enterococci included *En. durans* (1), *En. faecalis* (4), *En. faecium* (12), *En. hirae* (3), and *En. thailandicus* (2). Enterocin A (entA), enterocins mr10A and mr10B (mr10AB), and bacteriocin T8 (bacA) were the most commonly found structural genes in order of decreasing prevalence. Forty-five bacteriocin genes were identified within the 22 Bac+ isolates, each containing at least one of the screened structural genes. Of the 22 Bac+ isolates, 15 possessed two bacteriocin genes, seven isolates contained three different bacteriocins, and three isolates contained as many as four different bacteriocin genes. These results may explain the high degree of bactericidal activity observed with various Bac+ *Enterococcus* spp. Antimicrobial activity against wild-type *L. monocytogenes* and a bacteriocin-resistant variant demonstrated bacteriocins having different modes-of-action. Mixtures of bacteriocins, especially those with different modes-of-action and having activity against foodborne pathogens, such as *L. monocytogenes,* may play a promising role in the preservation of food.

## 1. Introduction

*Enterococcus* is a genus of Gram-positive, catalase-negative, facultative anaerobic cocci. Its members were removed from the genus *Streptococcus* in the 1980s because of DNA hybridization data showing that they were not closely related to streptococci and were moved into their own genus. The genus *Enterococcus* is now comprised of more than 20 species of which *En. faecalis* and *En. faecium* are the most common species and are readily found in the feces of mammals. Fecal enterococci have been classified as “fecal indicator” bacteria in much the same way as fecal coliforms, *i.e.*, as an indicators of unsanitary manufacture or processing of foods. Because of their association with animals and fecal distribution, enterococci are found worldwide in a large variety of fresh and prepared foods, vegetables, hard cheeses, meats and prepared meat products [1,2,3]. In France, *Enterococcus* spp. have been found in 44% of pasteurized-milk cheeses and as high as 92% of raw-milk cheeses [4]. They are present in numerous so-called “artisanal” food products that many are considered safe for consumption even though they are not generally regarded as safe (GRAS) by USDA regulatory approval. Although they have been involved in human enterococcal disease, they are considered opportunistic infections and account for a considerable amount of hospital-acquired nosocomial infections in the US [5]. Certain strains, however, are considered non-pathogenic and have been used as dietary adjuncts for reducing antibiotic-associated diarrhea in children [6,7]. In a comparative genomic study of probiotic *En. faecium* strain T-110, a non-pathogenic strain (NRRL B-2354), and three pathogenic strains, the non-pathogenic/probiotic strain was found to be lacking a number of virulence-related genes that were found in the pathogenic strains [8]. In an effort to define the desirable characteristics for *Enterococcus-*based probiotics, researchers have been examining genes in isolates from healthy individuals *vs.* those obtained as pathogenic strains from clinical isolates. Pathogenic strains of *Enterococcus* spp. exhibit a number of virulence factors including gelatinase, adhesion to collagen, aggregation substance, endocarditis antigen, and β-hemolytic substances which make them less likely candidates for use as food starter cultures [9]. 

*Enterococcus* spp. are also known to produce bacteriocins that have demonstrated antimicrobial activity against various foodborne pathogens [10,11]. Bacteriocins are ribosomally synthesized peptides produced by bacteria that are capable of killing other bacteria by forming pores in target membranes [12,13,14]. In lieu of issues with live cultures which may be consumed, the application of cell-free culture supernatants may be far less objectionable and find greater acceptance as food preservatives. This study examines the use of an *Enterococcus*-based PCR bacteriocin array to determine the presence of bacteriocin genes in 22 Bac+ *Enterococcus* strains isolated from food and animal sources having activity against *Listeria monocytogenes*.

## 2. Experimental Section

### 2.1. Bacterial Strains, Storage, and Growth Conditions

This study included 22 isolates of *Enterococcus* spp. of which twelve were *En. faecium*, four were *En. faecalis*, three were *En. hirae*, two were *En. thailandicus*, and one was *En. durans*. Frozen master cultures of enterococcal strains were stored in milk-based freezing media at −80 °C, and 100 μL of master culture was added to 10 mL of de Man, Rogosa, and Sharpe (MRS) broth at 30 °C for 12–16 h prior to use. Although MRS broth/agar would not usually be the initial choice for *Enterococcus* spp., they were initially isolated on MRS agar and grew well when cultured in MRS broth, as they were suspected of being traditional lactic acid bacteria until their eventual identification by 16S rRNA PCR and sequence analysis [15].

### 2.2. Activity against Listeria Monocytogenes 

**Figure 1:**
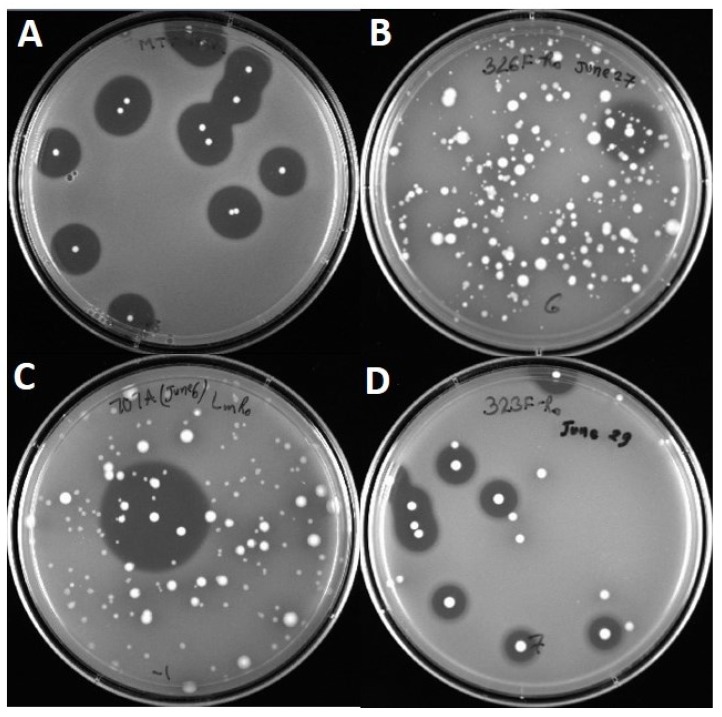
Inhibition of indicator lawns of *L. monocytogenes* 39-2 (R0) by various Bac^+^ *Enterococcus* spp. from platings of food or animal-related samples after enrichment. Panel A, *E. thailandicus* FS92; Panel B, *E. faecium* 326F; Panel C, *E. durans* FS707A; Panel D, *E. hirae* 323F.

All 22 strains of *Enterococcus* spp. were previously isolated from retail foods and animal sources as bacteriocin-producing enterococci as determined by overlay of ‘sandwiched’ colonies from plated samples (Figure 1) with indicator lawns containing *L. monocytogenes* 39-2 as described previously [15,16]. Subsequent analysis of specific strains was observed using culture spot assay against the wild type *L. monocytogenes* 39-2 (R0 strain) as well as a variant of *L. monocytogenes* 39-2 R0 that was isolated by selection against the bacteriocin produced by *Lactobacillus curvatus* FS47 (curvaticin FS47) and deemed “R1”. Inhibitory activity against *L. monocytogenes* 39-2 R0 (wild type) and R1 (curvaticin FS47^R^) was by Bac+ culture spot assay against *Listeria* indicator lawns.

### 2.3. Primer Creation

Bacteriocin genes were found using the online database Bactibase (Institut Supérieur des Sciences Biologiques Appliquées de Tunis, Tunis, Tunisia) [17] by searching for individual gene sequences produced by members of the genus, Enterococcus. Duplicates and highly homologous gene sequences were condensed into single primer sets. Gene selections were made using bacteriocin structural genes and neighboring immunity proteins to increase the size of the amplicon that would allow exclusion of homologous sequences. Adjacent ABC transporters and other similar features were excluded due to high homology between genes. Primers were designed from gene sequences using the online Primer3 software (Whitehead Institute for Biomedical Research, Cambridge, MA, USA) [18]. Once created, primers were analyzed against each bacteriocin gene using the MEGA 5.2 software (The Biodesign Institute, Tempe, AZ, USA) [19] to ensure cross-amplification would not occur. Primers were then ordered from Integrated DNA Technologies (IDT, Coralville, IA, USA).

### 2.4. PCR Detection of Enterococcal Bacteriocin Genes

The presence of bacteriocin related genes was determined by using 16 sets of primers in a PCR bacteriocin array as done previously [20]. Initially, total bacterial DNA was isolated using the BAX^®^ protease lysis method (DuPont Qualicon, Wilmington, DE, USA). Overnight culture (5 μL) was lysed in 200 μL protease mixture at 55 °C for 60 min followed by a deactivation step at 95 °C for 10 min. PCR amplification was performed on a DNA Engine Opticon 2 (MJ Research/Bio-Rad, Hercules, CA, USA) in 25 μL reaction mixtures. Each 25 μL reaction contained 12.5 μL iTaq™ Universal SYBR^®^ Green Supermix (Bio-Rad), 3.5 μL of each primer (final concentration of 60 nM for each primer), and 5 μL of the cell lysate diluted 1:5 with sterile, molecular grade water (DNAse-/RNAse-free). The cycling program was preceded by an initial denaturation at 95 °C for 15 min. The specific cycling parameters consisted of 40 cycles of the following: denaturation at 95 °C for 15 s, annealing at 60 °C for 60 s, and elongation at 72 °C for 60 s followed by a plate read. PCR products were verified by melting curve analysis (50 °C to 90 °C with a read every 0.2 °C and hold for 0.02 s) and electrophoresis on a 2% (mg/mL) agarose (FMC Corporation, Philadelphia, PA, USA gel at 80 V for 1 h, using a 100 bp ladder for size verification and viewed using the ChemiDoc™ XRS System UV transilluminator (Bio-Rad). PCR products were purified using the GenCatch™ PCR Cleanup Kit (Epoch Life Science, Missouri City, TX, USA) and submitted for sequencing to the Oklahoma State University Recombinant DNA and Protein Core Facility.

## 3. Results

### 3.1. Primer Design

Approximately 37 *Enterococcus* bacteriocin DNA sequences found in the Bactibase database were examined for designing primers. Alignment of the sequences in the database revealed that genes with identical sequences were often denoted as different bacteriocins with different accession numbers (Table 1). Upon grouping of highly homologous sequences, the DNA sequences were condensed to 16 unique enterococcal bacteriocin sequences listed in Table 1 with the names of highly homologous sequences cited under the heading “Homologous Genes”. Primary names for the primer pairs created with homologous genes were chosen subjectively.

### 3.2. PCR Detection of Enterococcal Bacteriocin Genes by Real-Time PCR

**Figure 2:**
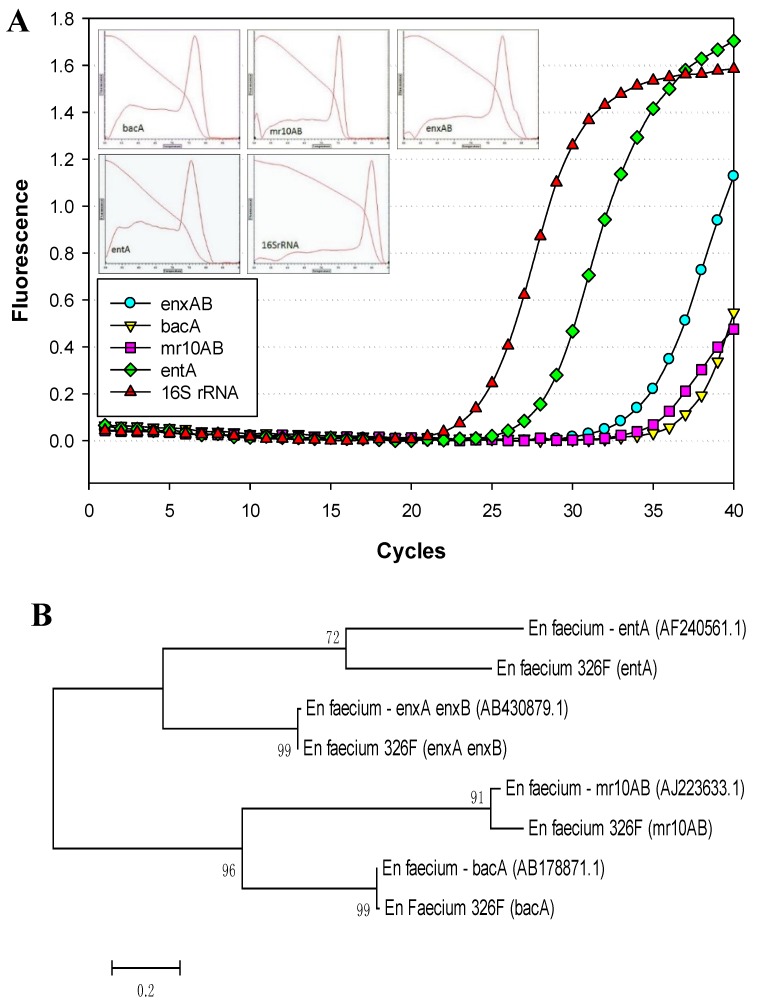
Real-time PCR and dendrogram of sequence alignment of four bacteriocins from *En. faecium* 326F. **Panel A**, real-time PCR amplification of *En. faecium* 326F using a PCR array of 16 primer pairs (in individual reactions) for known enterococcal bacteriocins, including a 16S rRNA control. Only positive amplification reactions are shown and include primers derived from enterocins enxAB, mr10AB, entA, bacA, and universal 16S rRNA; the 12 other primer sets did not amplify. Insets are for melting curve assays of the positive amplicons (as indicated). **Panel B**, maximum likelihood homology tree obtained from multiple sequence alignment and phylogenetic analysis of four bacteriocin-related sequences obtained with *En. faecium* 326F (entA, mr10AB, enxAB, bacA) *vs.* the GenBank bacteriocin sequences from which the primers were derived: AF240561.1 (entA), AJ223633.1 (mr10AB), AB430879.1 (enxAB), and AB178871.1 (bacA).

**Figure 3:**
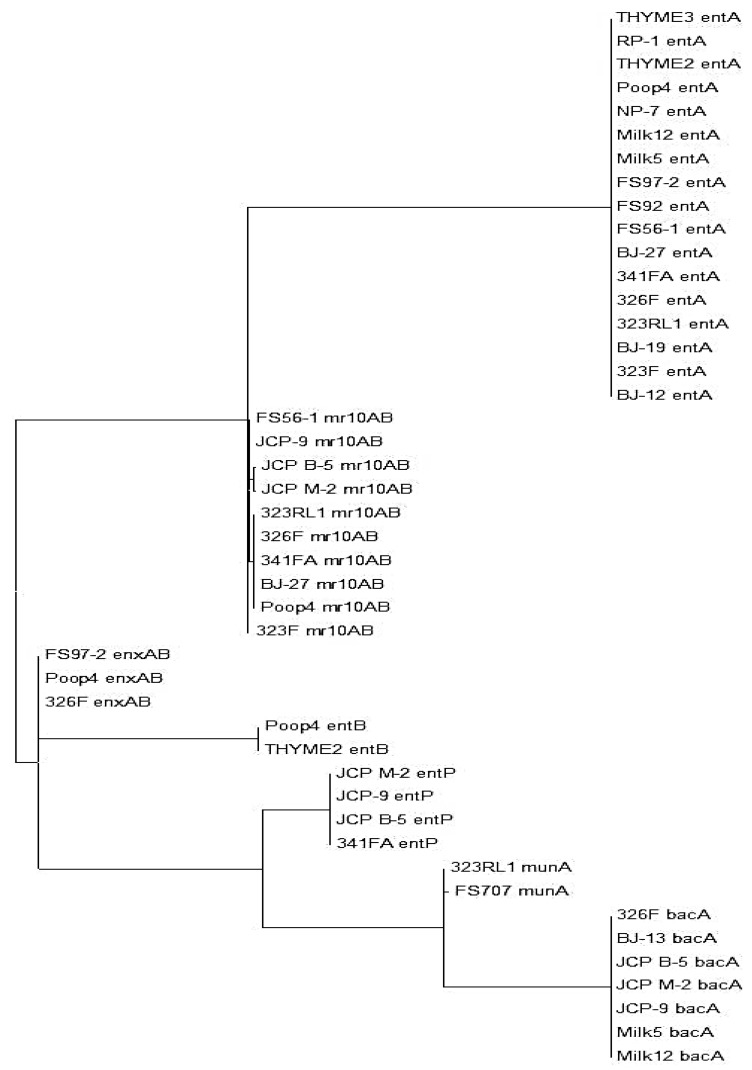
Maximum likelihood homology tree obtained from multiple sequence alignment and phylogenetic analysis of all bacteriocin-related sequences from the *Enterococcus* strains examined in this study. The bacteriocin name appearing after the strain designation is indicative of the enterococcal bacteriocin to which the amplimer had the highest homology.

The 22 *Enterococcus* isolates used in this study were pre-screened for the ability to inhibit *L. monocytogenes* (Figure 1) and we assumed they contained at least one bacteriocin gene [15]. The *Enterococcus* bacteriocin PCR array was obtained from previously sequenced bacteriocin genes from enterococci (as noted above), weeding out duplicates of those showing near-identical homology in order to reduce the number of primer sets in the “primer array” (Table 1). During analysis of our 22 isolates with all 16 sets of primers (*i.e.*, the “enterococcal PCR bacteriocin array”), the gene for the bacteriocin, enterocin A (entA), occurred most frequently among our isolates (77.3%, in 17 of 22 isolates) and was found among members of four of the five species examined in this study (Table 2). Bacteriocin mr10AB was the next most frequently detected bacteriocin among our isolates (63.6%). Values for homology relatedness for the partial sequences of various *Enterococcus* bacteriocins are presented in Table 2. Sequences obtained from our isolates using bacteriocin mr10AB primers were more highly homologous to enterococcin L50A and L50B sequences in the GenBank database than to other enterococcal bacteriocins because they are highly homologous (>95%) and were considered interchangeable in this study. Other enterococcal bacteriocin genes found within our isolates that showed high homology to known enterococcal bacteriocins include bacA (in seven of 22 isolates, 31.8%), enxAB (18.2%), entP (18.2%), entB (9.1%), and munA (9.1%). Many of the strains demonstrated the presence of more than one bacteriocin structural gene based on real-time PCR reactions (Table 2; Figure 2A). Sequence and phylogenetic tree analysis with the genes from which their primers were derived demonstrated four bacteriocin structural genes in *En. faecium* 326F (Figure 2B). Structural genes for the *Enterococcus* bacteriocins avicin A (avcA), columbicin A (colA), durancin Q (duqQ), enterocin 96 (ent96), enterocin C (entC), enterocin SE-K4 (entSE-K4), enterocins W α and β (enwAB), enterocin Q (entqA), and mundticin KS (munA) were not detected among our isolated strains. A maximum likelihood tree created from alignment of sequenced amplicons (using MEGA 5.2 software) show the distribution and similarity of bacteriocins among the *Enterococcus* isolates we examined (Figure 3).

### 3.3. Inhibition of L. monocytogenes 39-2 R0 (Wild-Type) and R1 (curvaticin FS47^R^)

**Figure 4:**
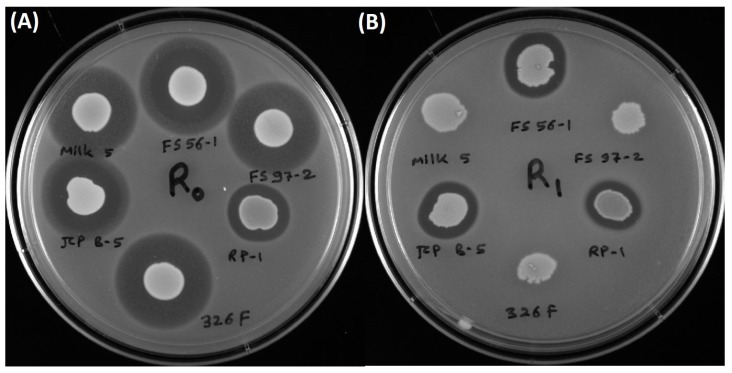
Culture spots of various Bac+ *Enterococcus* strains used in this study (*En. faecium* FS56-1, FS97-2, 326F, JCP B-5, Milk5; *En. thailandicus* RP-1) applied onto fresh lawns of wild type *Listeria monocytogenes* R0 (39-2) and a curvaticin FS47 Bac^R^ derivative of strain R0, *L. monocytogenes* R1 (39-2). Panel A, inhibition by all Bac+ culture spots against wild-type *L. monocytogenes* 39-2 R0 strain. Panel B, inhibition using the same cultures in panel A against the Bac^R^ *L. monocytogenes* R1 strain.

Inhibition of *L. monocytogenes* 39-2 (R0) was observed against by all *Enterococcus* strains tested as this was the indicator organism used in the original screening of samples from food and animal sources for anti-listerial Bac+ organisms (Figure 4) [15]. Inhibition of the Bacteriocin-resistant (Bac^R^) variant, *L. monocytogenes* 39-2 (R1), was only observed for approximately half the cultures showing inhibition against the wild type *Listeria* indicator strain (Figure 4). 

**Table 1:** Bacteriocin-related primer sequences used in this study.

Primer	Target Gene	Sequence (5′→3′)	Product Size (bp)	Included Genes	Homologous Genes
**1**	**Avicin A (avcA)**—FJ851402.1		236	avicin A precurson (avcA) immunity protein (avcI) divergicin-like bact (avcB)	sakacin X (sakX)
	Forward	ACG CGA AAT GAA GAA TGT TG			
	Reverse	TTT CAT TTC CGC CAG AAA AC			
**2**	**Columbicin A (colA)**—EF033111.1		299	columbicin A (colA) hypothetical protein (orfB)	bovicin A (bovA) enterocin AS-48
	Forward	TTT TTC TTG GGT TAT TTA CAG GAA			
	Reverse	ATG TGC AAT GGG CAA AAA CT			
**3**	**Durancin Q (duqQ)**—AB284369.1		384	immunity protein (duqI) durancin Q (duqQ) inducing peptide (duqF)	durancin TW-49 (durM)
	Forward	GCA CTG ATT CCG GCA CTA AT			
	Reverse	CGT AAC TCT AAT GGC GGG AAG			
**4**	**Enterocin 96 (ent96)**—FJ769024.1		291	enterocin 96	-
	Forward	GTG GAG AGG ACG AAA GGA GA			
	Reverse	TTG ATT AGT GGA GAG GAC GGT TA			
**5**	**Enterocin mr10A/mr10B (mr10AB)**		247	enterocin mr10A (mr10A) enterocin mr10B (mr10B)	enterocin JSB (entJSB) enterocin NA (entNA) enterocin NB (entNB) enterocin L50A (entL50A) enterocin L50B (entL50B) enterocin 62-6A (ent626A) enterocin 62-6B (ent626B) enterocin RJ-11
	Forward	ATG GGA GCA ATC GCA AAA T			
	Reverse	CAT CCT TGT CCG ATA AAC TGC			
**6**	**Enterocin C (entC)**—FU862242.1		506	enterocin C1 (entC1) enterocin C2 (entC2) enterocin C immunity (entCI)	enterocin 1081A enterocin 1071B
	Forward	AGG TCC AGC TGC TTA TTG GA			
	Reverse	CCA TTA GAA TGA ATA CGC TAA AGA AA			
**7**	**Enterocin SE-K4 (entSE-K4)**—AB092692.1		608	enterocin SE-K4 (entSE-K4) enterocin precursor (orf7) entSE-K4 homologue (orf8) entSE-K4 immunity (orf9)	bacteriocin II (D78257.1) enterocin TW-21 bacteriocin 31
	Forward	ATG TAG AAG CCG CCA CGT AT			
	Reverse	AAT CCC AAT CAT CCC ACA AA			
**8**	**Enterocin EJ97(ej97a)**—AJ490170.1		104	enterocin ej97	-
	Forward	AAA GCG ATG ATT AAG AAG TTT CC			
	Reverse	TCC CAA GGA TAA CGA CCG TA			
**9**	**Enterocin Wα/Wβ (enwAB)**—AB600897.1		423	enterocin W alpha (enwA) enterocin W beta (enw B)	-
	Forward	GGG GTT GAA TTA TTG TAG AAA GGA			
	Reverse	AAC TAG CCT CTA CCG CCA CA			
**10**	**Enterocin Q (entqA)**—DQ832184.1		231	enterocin Q (entqA)	-
	Forward	ATC ACA AAG TGA GCC CCT GT			
	Reverse	TGG TAT CGC AAA ATG GAT GA			
**11**	**Enterocin P (entP)**—AF005726.1		431	enterocin P (entP) enterocin P immunity (entQ)	-
	Forward	TTC CCC GAA GAA TAC AAA TGA			
	Reverse	AAT TTC TGG GGT GGC TAA TG			
**12**	**Enterocin A (entA)**—AF240561.1		362	enterocin A (entA) immunity protein (entI)	-
	Forward	AAA ATA AAT GTA CGG TCG ATT GG			
	Reverse	CCA GCA GTT CTT CCA ATT TCA			
**13**	**Enterocin B (entB)**—U87997,1		257	enterocin B (entB)	enterocin CRL35
	Forward	CAG AGT TCC CAA CTG TTT GCT			
	Reverse	AGC CCA TGC TAG TGG TCCT T			
**14**	**Enterocin Xα/Xβ (enxAB)**—AB430879.1		321	enterocin X alpha (enxA) enteorcin X beta (enxB)	-
	Forward	GGACAATTTATGGGTAAACAAGC			
	Reverse	TACGTCCACCATTCCAACCT			
**15**	**Bacteriocin T8 (bacA)**—AB178871.1		469	bacteriocin precursor (bacA) hypothetical immunity protein (bacB)	hiracin JM79 Bac43
	Forward	TTGTCTAGCTGGCATCGGTA			
	Reverse	CCAATAGAAGCCCATCCTCT			
**16**	**Mundticin KS (munA)**—KC291253.1		285	mundticin KS (munA)	mundticin L (munL) enterocin HF
	Forward	AAA AGG GTG CAG TGT TGA TTG			
	Reverse	TCC ACT GAA ATC CAT GAA TGA			

**Table 2:** *Enterococcus* strains used in this study and highest sequence homology ^1^ of partially sequenced bacteriocin operons to known bacteriocin operons.

Isolate	Species	entA	mr10AB	enxAB	bacA	entP	entB	munA
FS707	*En. durans*							99%
BJ-12	*En. faecalis*	100% ^2^						
BJ-13	*En. faecalis*				99%			
BJ-19	*En. faecalis*	100%						
BJ-27	*En. faecalis*	100%	96%					
326F	*En. faecium*	100%	92%	99%	99%			
FS56-1	*En. faecium*	100%	97%					
FS97-2	*En. faecium*	100%	99%	100%				
JCP B-5	*En. faecium*		97%		100%	99%		
JCP M-2	*En. faecium*		95%		100%	99%		
JCP-9	*En. faecium*		98%		100%	99%		
Milk12	*En. faecium*	100%	99%		100%			
Milk5	*En. faecium*	100%	99%	100%	99%			
NP-7	*En. faecium*	100%						
Pop4	*En. faecium*	100%	96%	100%			99%	
THYME2	*En. faecium*	100%					100%	
THYME3	*En. faecium*	100%						
323F	*En. hirae*	100%	97%					
323RL1	*En. hirae*	100%	96%					99%
341FA	*En. hirae*	100%	93%			99%		
FS92	*En. thailandicus*	100%	97%					
RP-1	*En. thailandicus*	100%						

^1^ Homology percentages based on highest “Max Score” by NCBI’s nucleotide BLAST program with our entire partial sequence obtained from sequencing.

## 4. Discussion

*Enterococcus* spp. are common inhabitants of mammalian intestinal flora and are often found among other lactic acid bacteria in the environment and isolated from foods. Similarly, our Bac+ enterococcal isolates represent a wide distribution of food and environmental samples such as fecal matter (323F, 326F, 341FA, Pop4), meat processing (JCP-9/B-5/M-2), dairy (Milk 5/12), rumen fluid (323RL1) and herbs (THYME 2/3). The 22 strains of *Enterococcus* spp. examined in this study had inhibitory activity against *L. monocytogenes* which was not attributed to bacteriophage, acid inhibition, or hydrogen peroxide and was eliminated by treatment with protease, complying with the classic definition of bacteriocins [15]. Enterococci have been well documented for the production of bacteriocins which may give them an environmental advantage against susceptible bacteria [21]. Bacteriocin-producing strains of *Enterococcus* have been isolated from fermented foods [22], the intestinal flora of mammals including humans [23], and retail raw and processed foods [16]. 

Although enterococci are part of the natural flora of the mammalian gastrointestinal tract, some strains produce virulence factors, are listed as BSL-2 strains, and can also be opportunistic causes of infection [22]. The presence of enterococci as part of the flora isolated from artisanal food products made from raw ingredients has been the subject of debate on whether they should be considered as food starter cultures [24] or allowed for use as surrogate organisms (that mimic pathogen survival) to define process conditions in food manufacturing facilities [25]. Some strains of *Enterococcus* have also been characterized for use as probiotics in commercial products, citing the absence of aggregation substance, collagen binding protein, hemolysins, resistance to reactive oxygen, and vancomycin resistance that could be troublesome in opportunistic infections [26]. Although there are successful applications of enterococcal probiotic cultures listed in the literature, FAO/WHO is still reluctant to give universal approval of *Enterococcus* as probiotic cultures based on potential acquisition and dissemination of antibiotic resistance determinants, notably vancomycin resistance [27].

The ability to produce multiple bacteriocins is a common feature among bacteriocinogenic lactic acid bacteria [21]. Liu *et al.* [9,28] found two bands of activity when they attempted to purify the bacteriocin produced by *En. faecium* LM-2, and further DNA sequencing identified both enterocin P and enterocin L-50 related structural genes in strain LM-2. Perez *et al.* [29] used electrospray ionization-liquid chromatography and mass spectrometry (ESI-LC/MS) to identify and monitor production profiles of four bacteriocins in culture supernatants of *En. faecium* NKR-5-3 that was isolated from fermented fish. Rehaiem *et al.* [22] used nine primer sets in PCR assays against *En. faecium* MMRA and found genes encoding four different enterocins. In this study, an *Enterococcus* bacteriocin real-time PCR array facilitated the identification of bacteriocin genes in all 22 Bac+ strains tested. The 16 primer sets used in this study (derived from 37 enterococcal bacteriocins) were employed in a bacteriocin PCR array that was successful in identifying not only individual bacteriocin structural genes in all 22 strains, but multiple bacteriocin genes in 15 of the 22 strains tested. DNA sequencing and sequence analysis confirmed that many of the multiple PCR reactions that occurred with any individual strain were due to different bacteriocins within each of the strains (Figure 2) whereby seven strains possessed one bacteriocin gene, five strains possessed two bacteriocins, seven strains possessed three bacteriocins, and three strains demonstrated the presence of as many as four different bacteriocins (Table 2; Figure 2 and Figure 3). The enterococcal bacteriocin PCR array defined herein is a quick and easy screen in future studies of bacteriocinogenic *Enterococcus* spp.

The presence of multiple bacteriocin genes suggests a potent antimicrobial mixture, however, it does not necessarily mean that all of them are produced in concert. Perez *et al.* [29] indicated that some of the multiple bacteriocins in *En. faecium* NKR-5-3 were not expressed simultaneously and that some regulated the expression of others. Although Liu *et al.* [9] used lengthy traditional biochemical protein purification techniques to identify multiple bacteriocins, and Perez *et al.* [29] used more sophisticated mass spectrometry, the identification of multiple enterococcal bacteriocin structural genes in this study was readily determined in a few hours by real-time PCR using an array of primers covering all known enterococcal bacteriocins (Table 2, Figure 4). Enterocin A was the most commonly found bacteriocin structural gene from the 22 *Enterococcus* isolates. Enterocin A shows sequence homology to class IIa bacteriocins such as the pediocin-like bacteriocins which are characterized for having antilisterial activity [30]. A high frequency of “pediocin-like” bacteriocins with antilisterial activity was expected among the Bac+ strains examined in this study since selection was biased on the use of *L. monocytogenes* as an indicator strain [31,32]. It is not clear if the spectrum of antilisterial activity demonstrated by these *Enterococcus* spp. is representative of enterococcal bacteriocins in general, as the distribution of Bac+ isolates was biased by the *Listeria* indicator used for their selection.

The ability to acquire resistance to LAB bacteriocins has been observed within *L. monocytogenes*. Naghmouchi *et al.* [33] observed cross-resistance in *L. monocytogenes* that was linked to changes in antibiotic sensitivity and cell wall palmitic acid content. Bacteriocin-resistant (Bac^R^) variants of *L. monocytogenes* were also used to identify same/different “modes-of-action” by various bacteriocins [34]. Bacteriocins that remained inhibitory to the Bac^R^ variant were considered to have a different mode-of-action than those which lost activity due to the resistance mutation [34]. Similarly, *En. faecium* Milk5, JCP B-5, FS97-2, FS56-1, 326F, and *En. thailandicus* RP-1 were all inhibitory against *L. monocytogenes* R0 (wild-type) but only JCP B-5, FS56-1, and RP-1 retained activity against *L. monocytogenes* R1 (Bac^R^; derived from R0) (Figure 4; Table 2). One reasonable explanation is that the elimination of activity by multiple bacteriocins was due to a common mode-of-action, or site-of-action, that was modified by the formation of the Bac^R^ mutation and the activity demonstrated by the remaining Bac+ strains is possibly due their action at a different site, or by a different mechanism (Figure 4B). Partial sequence analysis shows that *En. thailandicus* RP-1 possesses the structural gene for entA, *En. faecium* JCP B-5 possesses that for mr10AB, bacA, and entP, and *En. faecium* FS56 possesses entA and mr10AB, and all three strains are inhibitory to *L. monocytogenes* R1 (Bac^R^). However, both *En. faecium* Milk5 and 326F possess most of those genes and others (entA, mr10AB, enxAB, and bac3), yet lost activity against *L. monocytogenes* R1 (Table 2, Figure 4B). It is not clear what is involved to explain these differences in activity against *L. monocytogenes* R0 and R1. Different modes-of-action demonstrated by various bacteriocins have previously been shown to result in enhanced bacteriocin activity when mixed and this could be valuable information to use in further studies on the use of bacteriocins as antimicrobial preservatives in food [15,34].

The presence of *Enterococcus* spp. in numerous RTE food products, the commercial use of *Enterococcus* as probiotics, and the ability to produce inhibitors of pathogenic bacteria makes enterococcal bacteriocins potential candidates as natural food preservatives. Many of the concerns of *Enterococcus* as a probiotic is with the use of the live culture itself. However, the use of bacteriocin-containing cell-free culture supernatants as food preservatives may be less disconcerting than the use of viable bacteria. Further studies involving enterococcal bacteriocins, and the prospects of combining bacteriocins having different mode/site-of-action, as food preservatives may provide significant strides in the growing field of natural antimicrobials.

## 5. Conclusions

*Enterococcus* spp. carry many bacteriocin-related genes that provide antimicrobial activity capable of inhibiting competitors in a hypothetical ecological role, but may also be useful in activity against spoilage and pathogenic organisms in select food applications. Many strains encode more than one bacteriocin, although it remains to be seen which ones might be actively expressing bacteriocin proteins. The bacteriocin PCR array allowed quick and easy identification of the presence of bacteriocin-encoding genes and provided coverage for all known *Enterococcus* bacteriocins. Not only did the 22 strains have activity against *L. monocytogenes*, but the use of bacteriocin-resistant variants of *L. monocytogenes* allowed the identification of bacteriocins having different modes-of-action which may be useful in designing mixtures of bacteriocins that have synergistic activity against target organisms.
